# Long Non-coding RNAs and MicroRNAs Interplay in Osteogenic Differentiation of Mesenchymal Stem Cells

**DOI:** 10.3389/fcell.2021.646032

**Published:** 2021-04-09

**Authors:** Carmen Lanzillotti, Monica De Mattei, Chiara Mazziotta, Francesca Taraballi, John Charles Rotondo, Mauro Tognon, Fernanda Martini

**Affiliations:** ^1^Section of Experimental Medicine, Department of Medical Sciences, School of Medicine, University of Ferrara, Ferrara, Italy; ^2^Center for Musculoskeletal Regeneration, Houston Methodist Research Institute, Houston, TX, United States; ^3^Orthopedics and Sports Medicine, Houston Methodist Hospital, Houston, TX, United States; ^4^Laboratory for Technologies of Advanced Therapies, University of Ferrara, Ferrara, Italy

**Keywords:** long non-coding RNA, microRNA, crosstalk, osteogenic differentiation, mesenchymal stem cell, interplay, miRNA, lncRNA

## Abstract

Long non-coding RNAs (lncRNAs) have gained great attention as epigenetic regulators of gene expression in many tissues. Increasing evidence indicates that lncRNAs, together with microRNAs (miRNAs), play a pivotal role in osteogenesis. While miRNA action mechanism relies mainly on miRNA-mRNA interaction, resulting in suppressed expression, lncRNAs affect mRNA functionality through different activities, including interaction with miRNAs. Recent advances in RNA sequencing technology have improved knowledge into the molecular pathways regulated by the interaction of lncRNAs and miRNAs. This review reports on the recent knowledge of lncRNAs and miRNAs roles as key regulators of osteogenic differentiation. Specifically, we described herein the recent discoveries on lncRNA-miRNA crosstalk during the osteogenic differentiation of mesenchymal stem cells (MSCs) derived from bone marrow (BM), as well as from different other anatomical regions. The deep understanding of the connection between miRNAs and lncRNAs during the osteogenic differentiation will strongly improve knowledge into the molecular mechanisms of bone growth and development, ultimately leading to discover innovative diagnostic and therapeutic tools for osteogenic disorders and bone diseases.

## Introduction

Non-coding RNAs comprise a class of molecules without or with very low protein-coding capability. Indeed, most of the human genes are transcribed into RNAs, which are not translated into a protein (Li and Liu, 2019). In recent years, advances in high-throughput technologies and inter-and multi-disciplinary approaches have improved our knowledge in the field of non-coding RNAs and their complex signaling network in human cells. Long non-coding RNAs (lncRNAs) and microRNAs (miRNAs) are the two major families of non-protein coding transcripts that regulate a number of molecular mechanisms (Xue et al., 2017), including gene expression.

Gene expression regulation is involved in the control of different fundamental cellular processes (Rotondo et al., 2018b; Corazza et al., 2020; Oton-Gonzalez et al., 2021), including differentiation and proliferation (Ferronato et al., 2011; Rotondo et al., 2015, 2016, 2018a, 2020). Both lncRNAs and miRNAs have gained attention because of their activities as essential epigenetic regulators of gene expression in many tissues (Rotondo et al., 2013), including bone (Zhang X. et al., 2019). lncRNAs regulate gene expression through different mechanisms, acting both at transcriptional and post-transcriptional level (Fernandes et al., 2019). miRNAs regulate gene expression mainly by binding the mRNA target, inducing its degradation or translation inhibition (Oliveto et al., 2017).

Notably, lncRNAs and miRNAs exert their biological functions by forming a huge and complex regulatory network of mutual interactions leading to gene expression control. By this crosstalk, lncRNA and miRNA modulate cell differentiation, including the osteogenesis of mesenchymal stem cells (MSCs) (Wang L. et al., 2019; Sikora et al., 2020).

MSCs are promising competent biomedical candidates in the management of several bone disorders, as their use-availability (Iaquinta et al., 2019a; Mazzoni et al., 2020a). Indeed, MSCs own several important biological properties, such as the capacity to secrete molecules that can induce tissue regeneration, self-renewal and proliferation, as well as multipotentiality, anti-inflammatory and immunomodulatory effects (Pearson et al., 2015; Batta et al., 2016; Iaquinta et al., 2019a; Globig et al., 2020; Mazzoni et al., 2020b). Isolated for the first time from the bone marrow (BM) (Bianco et al., 2008), MSCs have been subsequently harvested from many different anatomical regions, such as adipose tissue (ASCs) (Mahmoudifar and Doran, 2015; Mazzoni et al., 2020a), umbilical cord (UC-MSCs) (Galderisi and Giordano, 2014), dental pulp tissues (DPSCs) (Ferro et al., 2014), and others (Fei et al., 2013; Ferro et al., 2014; Gong et al., 2014; Fierabracci et al., 2015). MSC osteogenic differentiation represents a key event in both natural bone healing, as well as in bone tissue engineering approaches (Barbanti Brodano et al., 2012; Manfrini et al., 2015; Barbanti Bròdano et al., 2017; Iaquinta et al., 2019a,b; Sprio et al., 2020; Mazzoni et al., 2021).

The aim of this review is to highlight the post-transcriptional interplay between lncRNAs and miRNAs as regulator of gene expression during the osteogenic differentiation of MSCs. Indeed, knowledge concerning functions of lncRNAs and miRNAs is rapidly growing by means of progress in new bioinformatics tools as well as experimental approaches (Grillone et al., 2020). Herein, we report data from *in vitro/in vivo* studies, which investigated the interaction between lncRNAs and miRNAs in osteogenic differentiation.

The studies included in this review were identified by a literature search in PubMed’s collection of articles that are related to lncRNA-miRNA network in osteogenesis. The combined search terms were “lncRNA” and “miRNA” and “osteogenesis” and “stem cells.”

## lncRNA and miRNA Functions

**FIGURE 1:**
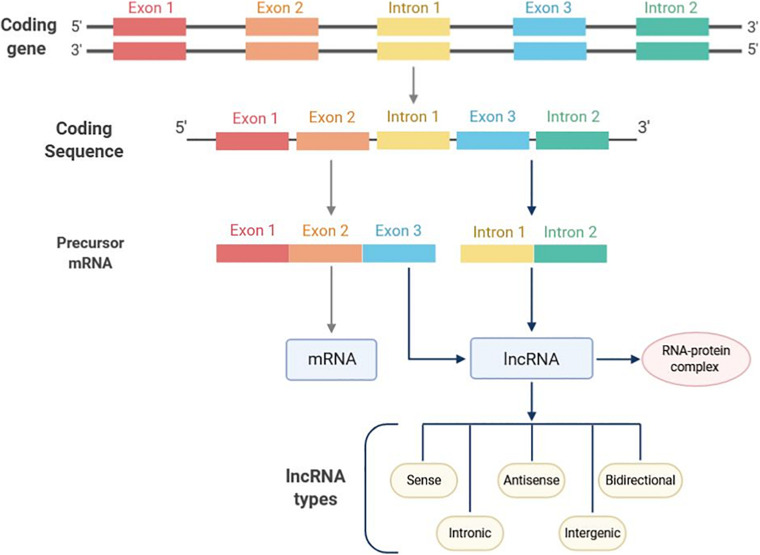
Long non-coding RNAs (lncRNAs) classification on the basis of localization. lncRNAs transcription mainly occurs in intronic regions. Enhancer and promoter regions can also undergo lncRNAs transcription. Based on the genomic position, lncRNAs can be divided into five categories, such as sense, antisense, bidirectional, intronic, and intergenic lncRNAs. Following biosynthesis, lncRNAs form a complex which includes a number interacting proteins to eventually sponge miRNA targets.

lncRNAs are a class of transcripts of over 200 nucleotides (nt) in length with absence or limited protein-coding potential (Chi et al., 2019). Discovered for the first time in mouse, lncRNAs can be localized in the nucleus, nucleolus and/or cytoplasm (Losko et al., 2016; Fernandes et al., 2019). In the mammalian genome, transcripts derived from lncRNAs are higher than those from protein-coding sequences representing 4–9% and 1% of the genome, respectively (Peng et al., 2018a). The biogenesis of lncRNAs is cell-type specific and their transcription can occur in different genomic regions, such as enhancers, promoters, and intergenic regions by the RNA-polymerase II enzyme (Dahariya et al., 2019). Subsequently, pre-mature lncRNAs are capped at the 5′-end with methyl-guanosine, polyadenylated at the 3′-end and generally expressed as alternatively spliced variants (Losko et al., 2016; Figure 1). The lncRNAs possess multiple activities based on different action mechanisms which include: (i) chromatin remodeling, (ii) blocking the promoter region of specific genes, (iii) regulation of transcription factors; (iv) forming double-stranded RNA complexes with mRNA or miRNAs, finally leading to gene expression regulation at both transcriptional and post-transcriptional levels.

lncRNAs participate in diverse biological processes, such as chromatin regulation and gene expression. In embryonic stem cells (Kouskoff et al., 2005), lncRNAs function as regulator of cell cycle, maintain the pluripotency, and also may be involved in the renewal and differentiation by interfering with molecular pathways during organogenesis (Aich and Chakraborty, 2020). Moreover, some studies highlighted different roles for lncRNAs in the regulation of bone formation from MSCs (Tye et al., 2018).

miRNAs are small RNA molecules of about 21–23 nt in length. They account for 1–5% (Macfarlane and Murphy, 2010) of the human genome and modulate approx. 30–60% of protein-coding genes (Muljo et al., 2010). In the genome, miRNA coding genes are frequently located within intergenic and intron regions (Olena and Patton, 2010) and are grouped in clusters where a single primary transcript generates multiple miRNA genes (Chang et al., 2015).

**FIGURE 2:**
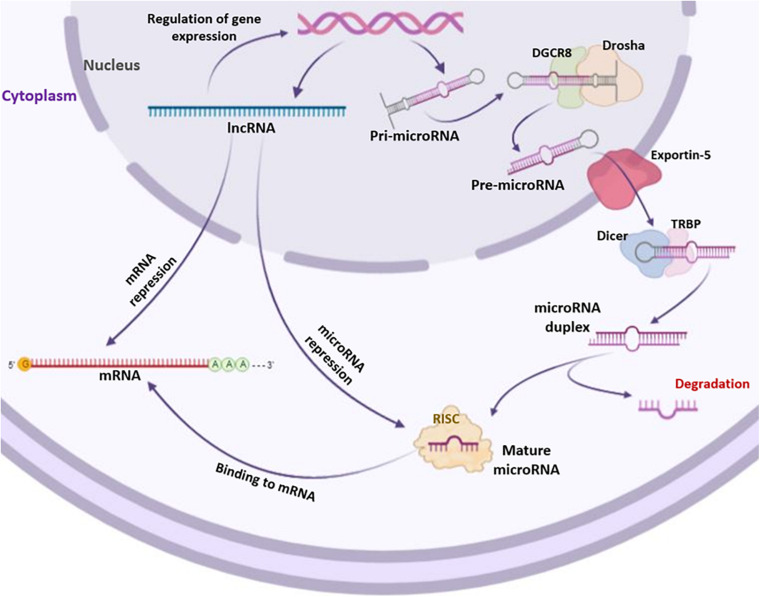
Biogenesis of long non-coding RNAs (lncRNAs) and microRNAs (miRNAs). lncRNAs and miRNAs are both transcribed by RNA polymerases II (Pol II). Transcribed lncRNAs directly bind mRNAs and/or miRNAs targets. Differently from lncRNAs, miRNAs undergo a series of processes to become mature. The primary miRNA precursor, i.e., pri-microRNA, is processed in pre-microRNA through the cutting activity of Drosha and DGCR8 enzymes. Then, pre-microRNA is transported into the cytoplasm by Exportin-5 where it is processed by Dicer and TRBP enzymes, forming microRNA duplex. The single strand of microRNA duplex is then complexed with RISC to act as formed mature microRNA by targeting mRNAs.

The generation of human miRNAs includes a two-step process that results in short RNA duplexes derived from long endogenous transcripts. The cleavages are performed by two ribonuclease III named Drosha and Dicer (Dexheimer and Cochella, 2020). The Drosha processes the pri-miRNA and generates the pre-miRNA, while the Dicer generates the miRNA duplex (Macfarlane and Murphy, 2010). From this duplex, one strand, called miRNA guide strand, is selected and assembled into the RNA-induced silencing complex (RISC). The activated RISC complex binds target mRNAs through base complementarity between the miRNA guide strand and the mRNA 3′UTR sequences (Dexheimer and Cochella, 2020; Figure 2). miRNAs regulate gene expression mainly by binding the mRNA target, inducing its degradation or translation inhibition depending on the degree of complementarity between the miRNA and the mRNA 3′-UTR; complete complementarity induces mRNA degradation, whereas partial complementarity inhibits mRNA translation. Gene coding regions, gene promoters or 5′-UTR may also represent binding sites for miRNAs (Oliveto et al., 2017). Most miRNAs are present inside the cells (Balatti et al., 2011). However, low levels of miRNAs are also found in extracellular environments, such as serum, plasma, blood, tears, urine, sperm, saliva, breast milk, and cerebrospinal fluid (Cai and Cullen, 2007; Hunter et al., 2008; Gallo et al., 2012; Moldovan et al., 2013; Rotondo et al., 2013; Bononi et al., 2016; Corral-Vazquez et al., 2017; Huang et al., 2018). Furthermore, miRNAs are also being studied as biomarkers of pathophysiological conditions (Bononi et al., 2016; Hoey et al., 2019) and as targets for innovative therapeutic approaches in precision medicine (Matin et al., 2016; Mazzoni et al., 2020b). Osteogenic differentiation can be regulated by several miRNAs both positively and negatively. In this context, miRNAs can act targeting either negative or positive regulatory genes and transcription factors (TFs) (Wang J. et al., 2019).

**FIGURE 3:**
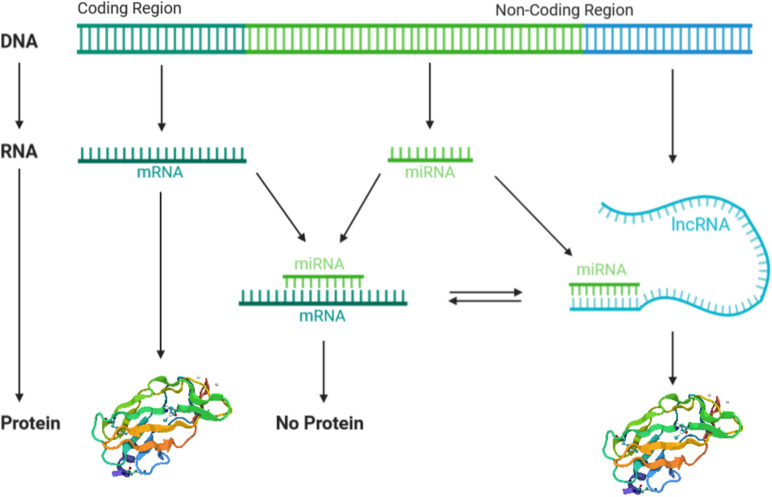
The crosstalk between long non-coding RNAs (lncRNAs) and microRNAs (miRNAs). lncRNAs and miRNAs are both transcribed from a non-coding region of the genome, meanwhile mRNAs are transcribed. lncRNAs compete with mRNAs to bind miRNA targets, by acting as sponge of miRNAs and thus abolishing the miRNA’s inhibitory action. miRNA–mRNA binding inhibits protein expression, whereas lncRNA–miRNA binding allows protein translation.

It is now established that lncRNAs and miRNAs activities are strictly interconnected in a complex manner. This regulatory network comprises two main aspects. One is the regulation of lncRNAs by miRNAs, while the other is the regulation of miRNAs by lncRNAs (Han et al., 2020; Sun et al., 2020). The first aspect provides the degradation of lncRNAs by miRNAs in an RISC/argonaute-dependent manner. In this case, miRNA binds to the target lncRNA 3′UTR, leading to the degradation of mRNA or blockade of the ribosomal machinery, both of which result in the negative regulation of gene expression (Sacco and Masotti, 2012). The second aspect, i.e., the regulation of miRNAs by lncRNAs, comprises different mechanisms. One mechanism provides the positive regulation of miRNAs by lncRNAs, where different miRNAs are splicing-dependent generated from the same lncRNA, which therefore acts as miRNAs precursor (Yoon et al., 2014; Zhou et al., 2021). In addition, lncRNAs can positively regulate the miRNA expression (Zhang et al., 2013), by forming chromatin remodeling complexes with enzymes involved in the histone acetylation and DNA methylation (Zhao et al., 2020). Furthermore, lncRNAs can interact as scaffolds, aiding the association of proteins with genomic DNA and promoting gene expression, including miRNA (Zhao et al., 2020). Finally, increasing evidence indicates that lncRNAs can negatively regulate miRNAs by competing with mRNA to bind miRNA-binding sites, leading to the regulation of miRNA target genes. In other words, lncRNAs work as competing endogenous RNAs (ceRNAs) by acting as sponge for miRNAs and abolish miRNA inhibitory action to the target mRNAs (López-Urrutia et al., 2019; Figure 3).

## MSCs Signaling Pathways in Osteogenic Differentiation

**FIGURE 4:**
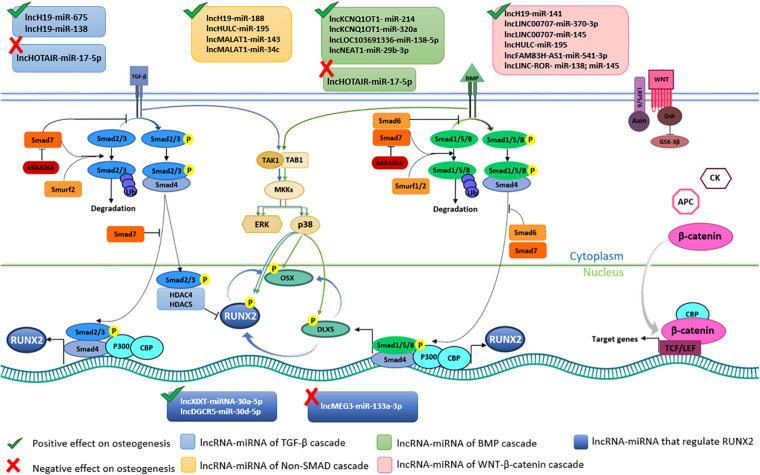
Long non-coding RNA (lncRNAs) and microRNAs (miRNAs) involved in osteogenic pathways in Bone Marrow Mesenchymal Stem Cells (BMSCs). Transforming growth factor-beta (TGF-β), bone morphogenic protein (BMP), and the Wingless/Int-1(Wnt)/β-catenin cascades are the pivotal pathways leading to BMSCs osteogenic differentiation. The binding between TGF-β and BMP with respective receptors activate SMAD-dependent and SMAD-dependent cascades. In TGF-β SMAD-dependent signaling, SMAD2/3 (R-SMAD) is phosphorylated upon ligan-receptor binding. Phosphorylated R-SMAD interacts with SMAD4 and translocate into the nucleus where, together with CBP and P300 co-activators, induce RUNX2 expression. In the cell nucleus R-SMAD without SMAD4 interacts with HDAC4/5 blocking RUNX2 expression. Unphosphorylated R-SMAD are degraded by ubiquitination. TGF-β SMAD-dependent pathways is positively regulated by lncH19-miR-675 and lncH19-miR-675, whereas it is negatively regulated by lncHOTAIR-miR-17-5p. In BMP SMAD-dependent cascade R-SMAD comprise SMAD1/5/8. SMAD6/7 and Smurf1/2 are negative regulators of this pathway. LncKCNQ1OT1-miR-214, lncKCNQ1OT1-miR-320a, lncLOC103691336-miR138-5p, lncNEAT1-miR-29b-3p trigger BMSCs differentiation. SMAD-independent signaling pathway induces DLX5, RUNX2 and OSX phosphorylation, which is favored by lncH19-miR-188, lncHULC-miR-195, lncMALAT1-miR-143, lncMALAT1-miR-34c axes. Wnt/β-catenin induces BMSCs osteogenic differentiation by β-catenin translocation into the nucleus and following expression of target genes. lncH19-miR-141, lncLINC00707-miR-370-3p, lncLINC00707-miR-145, lncHULC-miR-195, lncFAM83H-AS1-miR-541-3p, lncLINC-ROR- miR-138 and miR-145 positively regulate Wnt/β-catenin signaling pathway. lncXIXT-miRNA-30a-5p, lncDGCR5-miR-30d-5p directly positively regulate RUNX2, whereas lncMEG3-miR-133a-3p hamper RUNX2 expression.

MSCs differentiation into osteoblast lineage is a complicated network of key molecular/cellular actors, which lead to bone formation and remodeling (Thiagarajan et al., 2017). From a molecular point of view, MSC differentiation is mediated by several signaling pathways (Bhaskar et al., 2014). The two pivotal pathways are the Transforming Growth Factor-beta (TGF-β)/bone morphogenic protein (BMP) and the Wingless/Int-1(Wnt)/β-catenin pathways, which are strictly interconnected in a complex interplay (Liu J. et al., 2019; Figure 4). Although regulated by different factors, these two cascades converge on Runt-related transcription factor-2 (RUNX-2), which is the master transcription factor of the osteogenesis (James, 2013; Mevel et al., 2019). TGF-β is a cytokine belonging to the transforming growth factor superfamily (Poniatowski et al., 2015), whose production is mediated by white blood cells (Vallion et al., 2015). BMPs are a group of growth factors/cytokines, which have originally discovered for their ability to prompt bone/cartilage formations *in vivo* (Katagiri and Watabe, 2016). Over 20 BMP proteins have been identified so far. Specifically, BMP2, 4, 5, 6, 7, and 9 play a role in the osteogenic differentiation (Yang et al., 2014; Martini et al., 2020). TGF-β/BMP activate two different signal transduction pathways, that are Sma and Mad related proteins (SMAD)-dependent and -independent. Both signaling pathways convergently lead to osteogenic differentiation. During the SMAD-dependent cascade, different SMAD proteins are activated by phosphorylation. Receptor-phosphorylated/activated SMADs (R-SMADs) proteins includes SMAD2 and SMAD3 via TGF-β signaling and SMAD1, SMAD5 and SMAD8 via BMP pathway (Hata and Chen, 2016; Figure 4). R-SMADs from TGF-β/BMP pathways interact with SMAD4, that translocate into the nucleus complexed with SMAD2/3, thereby activating the expression of osteogenic genes, such as RUNX2 (Moghaddam and Neshati, 2019). On the other hand, TGF-β signaling pathway hampers the osteoblast differentiation by recruiting the histone deacetylases 4 and 5 (HDAC4/5) which inhibit the RUNX2 transcription (Kang et al., 2005; Thambyrajah et al., 2018). In Non-Smad-dependent cascade, TGF-β/BMP signaling pathways lead to the activation of the osteogenic TFs Osterix (OSX), Distal-less Homeobox 5 (DLX5) and RUNX2 (Chen et al., 2012; Komori, 2020), through the activation of the MAPK signaling cascade (Chen et al., 2012). Additional downstream genes involved in the osteogenic differentiation include osteopontin (OPN), osteocalcin (OCN), osteonectin (ON), and collagen type 1 (COL1) (Huang et al., 2007; Jonason et al., 2009). The Wnt/β-catenin signaling pathway plays a key role in MSCs proliferation, self-renewal and differentiation into osteoblast lineage, as it induces the expression of overlapping target genes that act synergistically (Mohammed et al., 2016). The Wnts consist in a large family of nineteen glycoproteins involved in a number of different biological processes, including osteogenic differentiation (Yang et al., 2016). β-catenin is a dual-function protein involved in cell adhesion and gene transcription (Valenta et al., 2012). MSCs osteogenic differentiation via Wnt/β-catenin dependent cascade leads to the expression of target genes involved in osteogenesis including *RUNX2* (Hamidouche et al., 2008; Aulicino et al., 2020; Figure 4).

TGF-β/BMP and Wnt/β-catenin signaling pathways have been shown to be connected (Rahman et al., 2015). Indeed, they can influence each other in a positive feedback loop, which allow the osteoblast differentiation *in vitro* and, therefore, a proper bone formation/remodeling *in vivo* (Dituri et al., 2019). Additional signaling pathways, such as the Parathyroid Hormone-related Peptide, NOTCH, Hedgehog, and Fibroblast Growth Factor have been found to be connected in a complex molecular network with TGF-β/BMP and Wnt/β-catenin signaling pathways upon osteogenesis (Chen et al., 2012; Rahman et al., 2015).

## Crosstalk Between lncRNA and miRNA in Osteogenic Differentiation of MSC

In recent years, the crosstalk between lncRNAs and miRNAs has emerged as a novel mechanism in the regulation of different cell molecular processes. lncRNAs can act as molecular decoys by sequestering miRNAs, and therefore inhibit miRNA negative interaction with the target genes (Wang L. et al., 2019). lncRNA/miRNA interaction allows correct function of the musculoskeletal system, controlling bone homeostasis and bone regeneration, as well as the osteogenic differentiation of stem cells (Sikora et al., 2020). Several lncRNAs have been involved in the osteogenic differentiation of MSCs. Relevant interactions between lncRNAs and miRNAs during the osteogenic differentiation of MSCs are reported below. Since most of the published literature relies on lncRNA/miRNA crosstalk in BMSC osteogenic differentiation, one section was specifically dedicated to these stem cells. One further section was reported for elucidating lncRNA/miRNA interaction in osteogenic differentiation of MSCs derived from different sources, such as embryo, adipose tissue, periodontal ligaments, and induced pluripotent stem cells.

## lncRNAs/miRNAs Crosstalk in Osteogenic Differentiation of Bone Marrow Mesenchymal Stem Cells

lncRNAs and miRNAs play an important role in regulating osteogenic differentiation of hBMSCs. Crosstalk between lncRNA and miRNA has been identified in different osteogenic pathways such as TGF-β/BMP-SMAD-dependent and -independent and Wnt/β-catenin signaling (Huang et al., 2015; Gong et al., 2018; Jiang et al., 2018). Specifically, the lncRNAs H19 (lncH19), KCNQ1OT1, nuclear-enriched transcript 1 (NEAT1), metastasis-associated lung adenocarcinoma transcript 1 (MALAT1), lncRNA LINC00707, lncRNA HULC, lncRNAs HOTAIR, maternally expressed gene 3 (lncRNA MEG3), XIXT and DGCR5 have been shown to play a crucial role as ceRNAs in promoting osteogenic differentiation of hBMSCs (Table 1).

**TABLE 1:** lncRNAs and their miRNA targets and competing mRNAs involved in MSCs osteogenic differentiation.

**lncRNA**	**miRNA target**	**Competing mRNA**	**Impact on osteogenesis**	**Examinated cell type**	**References**
lncH19	miR-141	SPAG9	Positive	SCAPs	Li Z. et al., 2017
lncH19	miR-138	PTK2	Positive	BMSCs	Wu J. et al., 2018
lncH19	miR-22 and miR-141	β-catenin	Positive	EMSCs	Gong et al., 2018
lncH19	miR-22 and miR-141	β-catenin	Positive	hMSCs BMSCs	Liang et al., 2016
lncH19	miR-17, miR-27b, miR34a, miR-106b, miR-107, miR-125a, miR-449a, and miR-449b	Notch pathway genes	Positive	iMAD	Liao et al., 2017
lncH19	miR-188	LCoR	Positive	mice BMSCs	Wang et al., 2018
lncMALAT1	miR-34c	SATB2	Positive	mice BMSCs	Yang et al., 2019
lncMALAT1	miR-30	RUNX2	Positive	hASCs	Yi et al., 2019
lncMALAT1	miR-143	OSX	Positive	hBMSCs	Gao et al., 2018
lncMALAT1	miR-124	RUNX2	Positive	C3H10T1/2	Zhang et al., 2020
lncPCAT1	miR-145-5p	TLR4	Positive	hASCs	Yu L. et al., 2018
lncPCAT1	miR-106a-5p	BMP2	Positive	PDLSCs	Jia et al., 2019a
lncMEG3	miR-27a-3p	IGF1	Positive	PDLSCs	Liu Y. et al., 2019
lncMEG3	miR-140-5p	RUNX2; OCN	Positive	hASCs	Li Z. et al., 2017
lncMEG3	miR-133a-3p	SLC39A1	Negative	BMSCs from PMOP	Wang et al., 2017
lncKCNQ1OT1	miR-214	BMP2	Positive	BMSCs	Wang C.-G. et al., 2019
lncKCNQ1OT1	miR-320a	SMAD5	Positive	BMSCs	Wang et al., 2020
lncKCNQ1OT1	miR-138	RUNX2	Positive	TSCs	Yu Y. et al., 2018
lncHOTAIR	miR-17-5p	SMAD7	Negative	BMSCs	Wei et al., 2017
lncLINC00707	miR-370-3p	WNT2B	Positive	BMSCs	Jia et al., 2019b
lncLINC00707	miR-145	LRP5	Positive	BMSCs	Cai et al., 2020
lncLINC02349	miR-25-3p	SMAD5	Positive	UC-MCSs	Cao et al., 2020
lncLINC02349	miR-33b-5p	WNT10B	Positive	UC-MCSs	Cao et al., 2020
lncLINC-ROR	miR-138; miR-145	ZEB2	Positive	BMSCs	Feng et al., 2018
lncHULC	miR-195	WNT and MAPK pathways genes	Positive	BMSCs	Jiang et al., 2018
lncFAM83H-AS1	miR-541-3p	WNT3A	Positive	BMSCs	Wu et al., 2020
lncLOC103691336	miR-138-5p	BMPR2	Positive	BMSCs	Li D. et al., 2019
lncNEAT1	miR-29b-3p	BMP1	Positive	BMSCs	Zhang Y. et al., 2019
lncRNA Rmst	miR-106, miR-125a, miR-449a; miR-449b	Notch pathway genes	Positive	iMAD	Zhang Z. et al., 2019
lncANCR/DANCR	miRNA-758	NOTCH2	Negative	PDLSCs	Peng et al., 2018b
lncTUG1	miR-204-5p	RUNX2	Positive	MSMSCs	Yu C. et al., 2018
lncXIXT	miRNA-30a-5p	RUNX2	Positive	hBMSCs	Zhang H.-L. et al., 2019
lncTCONS_00041960	miR-204-5p; miR-125a-3p	RUNX2	Positive	Rat BMSC	Shang et al., 2018
lncDGCR5	miR-30d-5p	RUNX2	Positive	BMSCs	Wu Z.-H. et al., 2018
lncNTF3-5	miR-93-3p	RUNX2	Positive	MSMSC	Peng et al., 2018c
lncMODR	miR-454	RUNX2	Positive	MSMSC	Weng et al., 2017
lncRNA-FER1L4	miR-874-3p	VEGFA	Positive	PDLSCs	Huang et al., 2020
lncRNA-POIR	miR-182	FoxO1	Positive	PDLSCs	Wang et al., 2016

The lncH19 is one of the most conserved non-coding transcripts that presents a significant role in promoting osteogenic differentiation (Li et al., 2020). LncH19 is largely expressed in the first stages of embryo development (Rotondo et al., 2013), while it is silenced in the majority cell types during adulthood. lncH19 activity as ceRNA has been reported in SMAD-dependent and Wnt/β-catenin signaling. A pro-osteogenic effect of lncH19 was shown in SMAD-dependent pathway via TGF- β1/Smad3/HDAC signaling in *in vitro* experiments. Moreover, additional data indicated that lncH19 induced bone formation *in vivo* (Huang et al., 2015). In particular, in Wnt/β-catenin signaling pathway, lncH19 sponged miR-22 and miR-141 inducing the upregulation of ALP, RUNX2, BMP and OCN and thus osteogenesis, whilst miR-22 and miR-141 hamper the osteogenesis binding the 3′-UTR and CDS regions of β-catenin (Liang et al., 2016). Further, lncH19 is also a positive regulator in tension-induced BMSC osteogenesis. In this context, lncH19 acts as a ceRNA of miR-138 for up-regulating the downstream focal adhesion kinase (FAK), which is a key factor in the mechano-transduction pathway of the osteogenic differentiation (Manfrini et al., 2013; Mazzoni et al., 2017; Wu J. et al., 2018). Finally, the ability of lncH19 to mediate the balance between osteogenic and adipogenic differentiation has been shown via miR-188 sponging. Once expressed, miR-188 negatively regulates the transcriptional corepressor ligand-dependent corepressor (LCoR), resulting in suppression of osteogenic differentiation and induction of adipogenic differentiation in murine BMSCs. Counter wise, lncH19 expression hampered the inhibitory effect of miR-188 on LCoR, leading to the osteogenesis (Wang et al., 2018).

Activation of the lncRNAs KCNQ1OT1, LOC103691336, NEAT1 and MALAT1 promoted osteogenic differentiation of BMSCs sponging miRNAs involved in BMP signaling pathway. The KCNQ1OT1 is an imprinted antisense lncRNA that can interact with different miRNAs (Wang C.-G. et al., 2019; Wang et al., 2020). On one hand, KCNQ1OT1, sponging miR-214 resulted in BMP2 expression during osteogenic differentiation (Wang C.-G. et al., 2019). Consistently, miR-214 silencing increased expression of the osteogenic genes SMAD1/5/8, RUNX2 and OSX, while KCNQ1OT1 inhibition completely inverted this expression pattern (Wang C.-G. et al., 2019). Also, KCNQ1OT1 sponging miR-320a, which targets SMAD5, resulted in SMAD5 expression during osteogenic differentiation of BMSCs, leading to the increased expression of a number of genes involved in osteogenesis, such as OCN, OPN, RUNX2 and ALP (Wang et al., 2020). LOC103691336 activated BMP cascade by sponging miR-138-5p in rat BMSCs, which led to the expression of BMPR2 (Li D. et al., 2019). Similarly, NEAT1 activated BMP cascade by sponging miR-29b-3p, resulting in BMP1 expression (Zhang Y. et al., 2019).

Differently from lncRNAs listed above, by sponging miRNAs, the lncRNAs HOTAIR and maternally expressed gene 3 (MEG3) negatively regulate the osteogenic differentiation of BMSCs. The biological functions of HOTAIR have been identified in human bone diseases (Wei et al., 2017). HOTAIR levels were found to be higher in samples of non-traumatic osteonecrosis of femoral head (ONFH) compared with osteoarthritis, and negatively correlated to miR-17-5p. Indeed, in BMP2-induced BMSC osteoblastic differentiation, HOTAIR reduced the expression of the osteogenic markers RUNX2, COL1A1, and ALP by binding miR-17-5p, which is a negative regulator of SMAD7 expression (Wei et al., 2017). Wang et al. (2017) reported that MEG3 inhibited the osteogenesis in hBMSCs positively regulating miR-133a-3p, which targets SLC39A1 gene encoding zinc transporter 1 (ZIP1) (Wang et al., 2017). However, both MEG3 and miR-133a-3p were increased in hBMSCs during postmenopausal osteoporosis, whereas markedly decreased in the differentiation of hBMSCs into osteoblasts. Therefore, the role MEG3 in osteogenesis remains to be clarified.

An additional study reported on an inverse correlation between the expression of MEG3 and miR-125b in MSCs cultured on the polymer poly(L-lactic acid); MEG3 and miR-125b have been found to be progressively upregulated and downregulated, respectively, throughout different culture time points. The same study also identified computationally four target sites for miR-125 binding within MEG3 gene, thus suggesting that this lncRNA might be under miR-125-mediated negative regulation (Izadpanahi et al., 2018). It has been reported that miRNAs can directly bind to the double stranded genomic DNA (dsDNA) by forming miRNA–dsRNA triple helices, thereby regulating the expression of genes (Paugh et al., 2016). However, to the best of our knowledge, the miRNA–dsRNA-mediated modulation of lncRNAs by miRNAs has not been described so far. In summary, since the lack of data, whether miRNAs might regulate osteogenic differentiation of MSCs by modulating lncRNAs remains to be assessed.

The Wnt/β-catenin cascade is positively regulated by the lncRNA LINC00707 during the osteogenic differentiation of BMSCs. By sponging miR-370-3p or miR-145, LINC00707 up-regulated WNT2B or LRP5 expression, respectively, which are two important players of Wnt/β-catenin pathway during osteogenic differentiation (Jia et al., 2019b; Cai et al., 2020). Furthermore, the pro-osteogenic effect of LINC00707 has been assessed also *in vivo*. Indeed, LINC00707 overexpression or silencing in BMSCs loaded on scaffolds and subcutaneously injected in mice, led to bone formation or impaired osteogenesis, respectively (Jia et al., 2019b). Similarly, the lncRNAs FAM83H-AS1 and Linc-ROR positively regulate the Wnt/β-catenin pathway by binding the negative regulators of osteogenesis miR-541-3p, and miR-138/miR-145, respectively. These lncRNA/miRNA interactions, resulted in inducing the expression of pro-osteoinductive proteins WNT3A and ZEB2 (Feng et al., 2018; Wu et al., 2020). Further, Wnt/β-catenin and p38MAPK pathway have been found to be enhanced by the lncRNA HULC upon sponging miRNA-195, which promoted proliferation and osteogenic differentiation of BMSCs (Jiang et al., 2018).

Irrespective of the osteogenic pathways, the lncRNAs XIXT and DGCR5 have been found to positively regulate BMSC osteogenic differentiation by directly acting on miRNAs targeting RUNX2, the master osteogenic transcription factor (Draper et al., 2016; Wu Z.-H. et al., 2018; Zhang H.-L. et al., 2019). XIXT and DGCR5 can trigger osteogenesis by sponging the miRNA-30a-5p and miR-30d-5p, respectively. Furthermore, the lncRNA TCONS_00041960, found downregulated in a microarray analysis of rat glucocorticoid-treated BMSCs, induced the expression of the osteogenic genes RUNX2 and GILZ by sponging miR-204-5p and miR-125a-3p, leading to enhanced osteogenesis (Shang et al., 2018). Finally, MALAT1, also known as nuclear-enriched transcript 2 (NEAT2), is a lncRNA emerging as modulator of osteogenesis in stem cells. In hBMSCs, MALAT1 is able to regulate OSX expression by sponging miR-143 leading to the osteogenic differentiation (Gao et al., 2018).

Recently, mouse models have been also used to investigate *in vivo* the role of the lncRNAs in osteogenic differentiation and their crosstalk with miRNAs. MALAT1 enhances osteoblast activity in osteoporotic animals inhibiting miR-34c, therefore promoting the expression of special AT-rich sequence-binding protein 2 (SATB2), a DNA-binding protein implicated in osteoblastic differentiation and skeletal development (Yang et al., 2019).

Few works studied the positive regulatory role of lncRNAs as miRNAs precursors during the osteogenic differentiation of MSCs. In particular, although it is known that lncH19 is a key positive regulator of osteogenesis by sponging a number of different miRNAs, this lncRNA can also exert a dual role in osteogenesis by generating three different miRNAs, i.e., miR-675, miR-675-5p and miR-675-3p. The first induces osteogenic differentiation in hBMSCs inhibiting (i) phosphorylation of SMAD3 by binding the TGF-β1 mRNA, and (ii) HDAC4/5 mRNA expression levels (Huang et al., 2015). Contrariwise, both miR-675-5p and miR-675-3p counteract osteoblast differentiation by targeting β-catenin transcript in hMSCs (Liang et al., 2016; Ma et al., 2020; Zhou et al., 2021).

Additional lncRNAs which may act as miRNAs precursors have been hypothesized (Song et al., 2015). An *in silico* analysis using high-throughput RNA sequencing data from MSCs identified the lncRNAs TCONS_00046478, TCONS_00027225, and TCONS_00007697 as precursors of miRNAs believed to be pro-osteogenic, i.e., miR-689, miR-544, and miR-640, respectively. However, functional studies need to be performed in order to identify the targets of these miRNAs and therefore their role in osteogenic differentiation (Song et al., 2015).

## Crosstalk Between lncRNAs and miRNAs in Osteogenic Differentiation of Mesenchymal Stem Cells From Other Sources

lncRNAs and miRNAs crosstalk has been shown to play a role also in the osteogenic differentiation of various stem cell types including those from ectomesenchyme, apical papilla, immortalized mouse adipose-derived mesenchymal stem cells (iMADs), adipose tissue, periodontal ligaments, maxillary sinus, umbilical vein cord and mouse embryo.

As shown in hBMSCs, in rat ectomesenchymal stem cells (EMSCs), lncH19 induced osteogenic differentiation via Wnt/β-catenin signaling pathway by binding the miR-22 and miR-141, leading to up-regulation of ALP, RUNX2, BMP and OCN (Liang et al., 2016; Gong et al., 2018).

Unlike hBMSC, in stem cells from apical papilla (SCAPs) lncH19 positively induced osteo/odontogenic differentiation through SMAD-independent signaling (Li Z. et al., 2019). Specifically, lncH19 competitively bound to miR-141 and prevented sperm-associated antigen9 (SPAG9) from miRNA-mediated degradation in SCAPs, thus increasing phosphorylated levels of p38 and JNK and inducing the expression of several osteogenic markers such as OCN, OSX, RUNX2, and ALP (Li Z. et al., 2019). In iMADs, during BMP9-induced osteogenic differentiation lncH19 functions as an important mediator of BMP9 signaling by modulating Notch signaling-targeting miRNAs, such as miR-17, miR-27b, miR34a, miR-106b, miR-107, miR-125a, miR-449a, and miR-449b (Liao et al., 2017). Notably, the impact of lncH19 on bone formation has also been demonstrated *in vivo* via mice transplantations of SCAP and iMAD cells co-transfected with this lncRNA (Liao et al., 2017; Li Z. et al., 2019).

An interplay between the lncRNA Rmst (lncRmst) and four miRNAs, miR-106, miR-125a, miR-449a, and miR-449b, has been shown in BMP9-induced iMADs osteogenic differentiation. These four miRNAs specifically target mRNAs of Notch receptors and/or ligands preventing Notch signaling activation and thus MSCs osteogenic differentiation. In addition, *in vivo* data confirmed the positive effect of lncRmst on osteogenesis. Indeed, lncRmst knockdown in iMADs subcutaneously injected in nude mice decreased BMP9-induced bone formation (Zhang Z. et al., 2019). Overall, these results indicate that the lncRNA-miRNA-Notch regulatory axis may play an important role in mediating BMP9-induced osteogenic differentiation of MSCs.

In human adipose stem cells (hASCs), MALAT1 and the prostate cancer-associated ncRNA transcript-1 (lncPCAT1) are able to positively regulate RUNX2 expression and the Toll-like receptor (TLR) signaling pathway by sponging miR-30 and miR-145-5p, respectively, leading to the osteogenic differentiation (Yu L. et al., 2018; Yi et al., 2019). Finally, sponging or not miR-140-5p, the lncMEG3 could regulate the balance between osteogenic and adipogenic differentiation of hASCs (Li Z. et al., 2017). Indeed, MEG3 up-regulation inhibited miR-140-5p leading to osteogenesis via RUNX2 and OCN expression, whereas MEG3 down-regulation led to adipogenesis. The implantation of hASCs with silenced MEG3 in nude mice confirmed the pro-osteogenic effect of this lncRNA *in vivo* (Li Z. et al., 2017).

In periodontal ligament stem cells (PDLSCs), largely studied especially for periodontal regeneration, the lncRNAs PCAT1 and MEG3 positively regulated osteogenesis sponging miRNAs. Specifically, *in vitro* results indicated that PCAT1 up-regulates BMP2, which is a direct target of miR-106a-5p and its transcription factor E2F5 expression sponging miR-106a-5p. *In vivo*, the transplantation of scaffolds loaded with PDLSC cells overexpressing/silencing lncPCAT1 in nude mice increased/reduced osteogenesis, respectively (Jia et al., 2019a). In the same cells, upon miR-27a-3p sponging, MEG3 promoted osteogenic differentiation activating IGF1-induced PI3k/Akt signaling pathway, which was hampered in periodontitis (Liu Y. et al., 2019). On the contrary, the anti-differentiation non-coding RNA (ANCR) negatively controlled osteogenesis in PDLSCs sponging miR-758, which is a negative regulator of NOTCH2 expression. In conclusion, lncRNA-ANCR/miR-758/Notch2-Wnt/β-catenin signaling pathway was suggested to be involved in the process of regulating osteogenic differentiation of PDLSCs (Peng et al., 2018b). An additional study carried out in PDLSCs reported that lncRNA Fer-1-like family member 4 (FER1L4) promotes osteogenic differentiation by binding miR-874-3p, which targets vascular endothelial growth factor A (VEGFA). The pro-osteogenic effect of lncRNA FER1L4 has been shown in mice transplanted with PDLSCs overexpressing the FER1L4 (Huang et al., 2020). A microarray analysis of PDLSCs from periodontitis patients, reported the dysregulation of the lncRNA-POIR. It has been reported that *in vitro* and *in vivo*, lncRNA-POIR positively regulates PDLSCs osteogenic differentiation and prompts bone formation, respectively. Specifically, in PDLSC cells lncRNA-POIR exerts its pro-osteogenic potential by sponging miR-182, which targets Forkhead box protein O1 (FoxO1) involved in the Wnt/β-catenin signaling cascade (Wang et al., 2016).

In maxillary sinus stem cells (MSMSCs), the lncRNAs TUG1, lncNTF3-5 and MODR positively regulated osteogenic differentiation sponging miRNAs targeting RUNX2, such as miR-204-5p, miR-93-3p, and miR-454, respectively (Weng et al., 2017; Peng et al., 2018c; Yu C. et al., 2018). Moreover, the positive regulation of lnc-NTF3–5 in osteogenesis has been demonstrated in mice through subcutaneous implantation of MSMSCs co-transfected with lncNTF3-5 and its target miR-93-3p (Peng et al., 2018c).

Umbilical cord-derived mesenchymal stem cells (hUC-MSCs) have been employed to study lncRNA LINC02349 effect on osteogenesis. During the osteogenesis of hUC-MSCs, lncRNA LINC02349, highly expressed during cell differentiation, affected WNT/β-catenin and BMP pathway up-regulating SMAD5 and WNT10B via inhibition of miR-25-3p and miR-33b-5p activities, respectively (Cao et al., 2020). The overexpression of LINC02349 in hUC-MSCs loaded on collagen scaffolds and subcutaneously implanted into nude mice, confirmed the positive effect of this lncRNA on osteogenic differentiation *in vivo* (Cao et al., 2020).

In mouse embryos derived C3H10T1/2 cells, MALAT1 promoted the osteogenic differentiation by sponging miR-124. Consistently, the expression of MALAT1 corresponded with a lower expression of miR-124 and an increased expression of pro-osteogenic genes such as RUNX2, OPN, and OCN (Zhang et al., 2020).

The role of the lncKCNQ1OT1 in the osteogenic differentiation of tendon stem cells (TSCs) has been investigated in a tendon injury mouse model. The study revealed that KCNQ1OT1 sponged miR-138 allowing RUNX2 expression and thus osteogenic differentiation of TSCs (Yu Y. et al., 2018).

## Conclusion

The discovery of the interplay between lncRNAs and miRNAs has improved knowledge into the gene expression regulation involved in bone development, bone homeostasis and bone regeneration. The lncRNAs activity as ceRNAs to suppress miRNA inhibitory effect is currently the main molecular mechanism investigated in osteogenic differentiation. Overall, the studies mentioned in this review clearly demonstrate that lncRNAs are essential modulators of the differentiative processes and finely coordinate the osteogenic differentiation of MSCs through crosstalk with miRNAs. Moreover, lncRNAs and miRNAs interplays may exert positive or negative effect on MSCs osteogenic differentiation.

As the biogenesis and expression of lncRNAs are cell-type specific, they may be ideal markers and therapeutic targets (Grillone et al., 2020). In this view, stem cells from bone marrow display useful biological properties for use in clinical practice and tissue regeneration. Natural bone healing and repair is essentially based on the activities of MSCs, mainly BMSCs. Further, MSCs are currently considered as valid biological sources for use in bone tissue regeneration by tissue engineering approaches. Therefore, deeply knowledge of the mechanisms which govern BMSCs differentiation is of pivotal relevance. Currently, different lncRNA/miRNA axes have been found positive regulator of osteogenesis in BMSCs, such as lncH19/miR-675, lncH19/miR-22-miR-141, lncH19/miR-138, lncH19/miR-188, KCNQ1OT1/miR-214, KCNQ1OT1/miR-320a, LOC103691336/miR-138-5p, NEAT1/miR-29b-3p, MALA-T1/miR-143, lncRNA LINC00707/miR-370-3p, LINC0 0707/miR-145, FAM83H-AS1/miR-541-3p, Linc-ROR/miR-138-miR-145, HULC/miRNA-195, XIXT/miRNA-30a-5p, DGCR5/miR-30d-5p, TCONS_00041960/miR-204-5p, TCONS_ 00041960/miR-125a-3p (Huang et al., 2015; Liang et al., 2016; Wang et al., 2017, 2018, 2020; Wei et al., 2017; Feng et al., 2018; Gao et al., 2018; Jiang et al., 2018; Shang et al., 2018; Wu J. et al., 2018; Wu Z.-H. et al., 2018; Jia et al., 2019a; Li D. et al., 2019; Wang C.-G. et al., 2019; Zhang H.-L. et al., 2019; Zhang Y. et al., 2019; Cai et al., 2020; Wu et al., 2020). By contrast, HOTAIR/miR-17-5p and MEG3/miR-133a-3p have been found to be negative regulators of BMSCs osteogenesis (Wang et al., 2017; Wei et al., 2017).

MSCs can be easily collected from other different anatomical regions, such as adipose tissue and apical papilla of the tooth, and also be valid biological sources for therapeutic application in orthopedic and endodontic clinical practice of bone tissue regeneration (Kolaparthy et al., 2015). Although the osteogenic/odontogenic differentiation of ASCs and SCAPs has been proven in several studies, only few reported on the crosstalk between lncRNAs and miRNAs during the osteogenesis of these MSCs.

In addition to the direct post-transcriptional regulation of miRNAs by lncRNAs, these two molecules can form a complex regulatory network of mutual interaction for controlling gene expression, which covers different mechanisms and a variety of molecular factors. However, the role of these additional mechanism on the osteogenic differentiation of MSCs has been poorly investigated so far. The understanding of these mechanisms upon osteogenic differentiation of MSCs will increase our knowledge of this biological process thereby leading to improved medical treatments of bone related diseases.

In conclusion, in this review we have described the current knowledge on the interplay between lncRNAs and miRNAs during the osteogenic differentiation of MSCs. Most of the works mentioned above have been conducted in BMSCs, thus further studies on MSCs collected from other anatomical districts are encouraged. Notably, some *in vivo* studies begin to confirm the biological role of lncRNAs molecular functions. Certainly, we can foresee a wider influence of lncRNAs on cell differentiation, because of the different molecular activities of lncRNAs, which largely remain to be clarified yet (Statello et al., 2020). We believe that recent discoveries on lncRNAs/miRNAs crosstalk upon osteogenic differentiation described herein will improve knowledge into the molecular mechanisms of bone remodeling and bone-related disorders, as well as will prompt the development of novel preclinical and clinical studies, ultimately leading to novel therapeutic approaches in the treatment of osteogenic disorders and bone diseases.

## Author Contributions

CL, CM, and JR selected the literature, designed the figures, and wrote the first draft of the manuscript. FT, MD, MT, and FM organized and supervised the work, and corrected the manuscript draft. MD, MT, and FM wrote the final version of the manuscript. All authors contributed to the article and approved the submitted version.

## Conflict of Interest

The authors declare that the research was conducted in the absence of any commercial or financial relationships that could be construed as a potential conflict of interest.
